# Effect of lifelong sucrose consumption at human-relevant levels on food intake and body composition of C57BL/6N mice

**DOI:** 10.3389/fnut.2022.1076073

**Published:** 2022-12-15

**Authors:** Ruolin Yan, Vivian Wai Wan Choi, Tania Hartono, Iris Mei Ying Tse, Margaret Chui Ling Tse, Yunpeng Zhou, Jinfeng Xu, Wai Hung Sit, Jennifer Man Fan Wan, Edmund Tsz Shing Li, Chi Bun Chan, Jimmy Chun Yu Louie

**Affiliations:** ^1^Faculty of Science, School of Biological Sciences, The University of Hong Kong, Pok Fu Lam, Hong Kong SAR, China; ^2^School of Biomedical Sciences, Li Ka Shing Faculty of Medicine, The University of Hong Kong, Pok Fu Lam, Hong Kong SAR, China; ^3^Department of Statistics and Actuarial Sciences, Faculty of Science, The University of Hong Kong, Pok Fu Lam, Hong Kong SAR, China

**Keywords:** sugar, lifelong, food intake, body composition, obesity, glycemia, lipid, life expectancy

## Abstract

**Introduction:**

Controversies surround the issue if chronic consumption of a high-sugar diet is detrimental to health or not. This study investigates whether lifelong consumption of a higher sucrose diet will induce overeating, and obesity, and cause metabolic dysfunctions such as hyperglycemia and dyslipidaemia in C57BL/6N mice, compared to a lower sucrose diet.

**Methods:**

Male C57BL/6N mice at 3 weeks of age were randomized into consuming a diet with 25 or 10% kcal from sucrose for the rest of their lives. Body weight, food and water intake, fasting blood glucose, insulin, and lipid levels were measured at regular intervals. At the end of the study, organs and tissues were collected and gene expression was measured.

**Results:**

There was no discernible difference in the impact on food intake, body composition, glucose and lipid homeostasis, liver triglyceride content, life expectancy, as well as gene expression related to intermediary metabolism between mice fed a diet with 10 vs. 25% kcal as sucrose over their lifespan. We also showed that switching from a 25% kcal diet to a 10% kcal diet at different life stages, or vice versa, did not appear to affect these outcomes of interest.

**Discussion:**

The results from our study suggest that lifelong consumption of a higher sugar diet generally did not induce overeating and obesity, disrupt carbohydrate metabolism and lipid homeostasis, and reduce life expectancy compared with a lower sugar diet. Our unorthodox findings disagreed with the popular belief that higher sugar consumption is detrimental to health, which should be confirmed in future studies.

## 1 Introduction

The recently updated guidelines for sugar consumption from the World Health Organization recommend that consumption of free sugars should be limited to less than 10% of total energy intake (1), as high free sugars consumption has been linked to higher risks of obesity, cardiovascular disease (CVD), and non-alcoholic fatty liver disease (NAFLD) (1, 2). Populations at all life stages generally consume more added and free sugars than that recommended by dietary guidelines (3). Some individuals even consume free sugars at as high as 40–50% of total energy intake (4). Obesity is a risk factor for various metabolic and endocrine abnormalities, such as hyperglycemia, hypertension, and dyslipidemia (5, 6). High regular sugar consumption has been claimed to be obesogenic by inducing overeating and body weight gain (7). However, whether high sugar consumption is detrimental to health remains a controversial topic (3, 8), as some researchers suggest that sugars are merely a source of calories similar to proteins and fats (9, 10), while *null* findings have also been reported in some clinical and observational studies (8, 11, 12).

Free sugars in the food supply mainly exist in two forms: sucrose or table sugar and high fructose corn syrup (HFCS). Sucrose is a disaccharide, consisting of 50% glucose and 50% fructose linked covalently, popular in Australia and other countries (13); HFCS, on the other hand, contains approximately 55% fructose and 45% glucose, that are not linked together, and is popular in the US (13). The fructose moiety of sucrose and HFCS has been suggested to be the culprit of potential detrimental health effects associated with high sugar intake (14, 15), such as insulin resistance, glucose intolerance, hyperglycemia, and hypertriglyceridemia in animals in the short-term (16, 17), as well as reduced lifespan and fecundity (17–20). However, some researchers have suggested that high fructose/sugar intake may not cause overeating, obesity, hepatic lipid accumulation, dyslipidemia, and hyperinsulinemia (21–23).

While conclusions from a variety of animal studies generally support the adverse health effects of high sugar consumption, several important limitations exist, including the use of fructose alone as the treatment and failure to include a control group where only glucose is consumed (9), administration of a supraphysiological dosage of sugars, as well as the unrealistic use of starch as the control diet that is devoid of sugars (24). The latter is of particular importance as an inappropriate control diet is likely to lead to incorrect conclusions or at least an inaccurate assessment of the effect size (25). Moreover, most of the studies that show adverse effects of high sugar consumption on mortality and lifespan were conducted on invertebrate animals such as *C. elegans* and *D. melanogaster*, which does not readily translate into the human situation, thus studies that examine such relationship in vertebrate animals are necessary. In addition, there is little evidence regarding the impact of chronic high sugar consumption on health outcomes. Mice have generally been used to identify the culprit of potential detrimental health effects associated with high sugar intake (9), as their genomes and organ system are similar to that of humans, and they develop diseases in a comparable way to humans (26). Although they do differ from humans in intermediary metabolism (27), which may undermine the translatability of rodent findings to humans (28), their short lifespan makes it an ideal model to feasibly study the lifelong effect of diets.

To address this important research gap, we conducted a study in male C57BL/6N mice to test the hypothesis that lifelong consumption of a higher (25% kcal) sugar diet is more likely to induce overeating, obesity, impaired glucose metabolism and lower life expectancy compared to a lower (10% kcal) sugar diet. It should be noted that 25% kcal is very close to the upper end of the range of free sugar intake typically found in modern societies as defined by mean + 1 standard deviation (SD) (3), and lower than that of some individuals with extreme intakes (4).

## 2 Materials and methods

### 2.1 Animals and dietary interventions

This study is a lifelong, longitudinal study. While there are strain variations of metabolic response when the mice are challenged by diet intervention, C57BL/6 mice displayed comparable responses to diet-induced obesity as other stains such as 129×1, DBA/2, and FVB/N (29). The C57BL/6N strain mice were chosen because they are susceptible to diet-induced obesity, type 2 diabetes and atherosclerosis (30), and is one of the most commonly used models in studying the effects of diet-induced metabolic disturbances [e.g., (31)]. Yet unlike the C57BL/6J strain, which is also commonly used for diet-induced obesity studies, the BL/6N strain does not have the naturally occurring in-frame five-exon deletion in *Nnt* (32–34). Therefore, compared to C57BL/6J strain, C57BL/6N strain is a better choice for the current study which examines the normal physiological responses to chronic consumption of dietary doses of sucrose to enhance translatability to humans. Male C57BL/6N mice (*n* = 20, the minimum number required for meaningful statistical analysis) were obtained from the Laboratory Animal Unit of The University of Hong Kong at 3 weeks of age, and data collection continued until their natural death, the experimental humane endpoint or 104 weeks of age, whichever was earlier. Female mice were not included to minimize the effect of hormone fluctuations on study outcomes. The experimental humane endpoint was defined as weight loss of ≥15% of peak body weight.

The mice were randomized into 2 groups using computer-generated random numbers (*n* = 10 each) as illustrated in Table 1. Every two mice were housed in one cage with a cage card for identification purposes but separated using custom-made cage covers that divides the cage in half so that they could smell and see each other to maintain wellbeing, while at the same time allowing for individual assessment of food and water intake (Supplementary Figure 1). They were kept in a 12-h light/dark cycle under SPF conditions. To facilitate the identification of mice in the same cage, one of the mice was hole-punched in its left ear. The health status/wellbeing of the mice was checked daily to ensure on-time detection of any signs of suffering and illness, as well as cases of sudden death of the mice. All procedures were performed in accordance with relevant laws and institutional guidelines. The ARRIVE guidelines have also been followed. The study was approved by the Committee on the Use of Live Animals in Teaching and Research of the Laboratory Animal Unit, The University of Hong Kong (Ref. #: 4104-16).

**TABLE 1:** Dietary intervention of groups at different life stages.

		Life stage				
		
		Early childhood	Childhood to adolescence		Early adulthood	Late adulthood
					
Group	*n*	(3–6 weeks)	(6–9 weeks)	(9–12 weeks)	(12–72 weeks)	(72–104 weeks)
10% kcal	10	Control G^a^	Control G	Control^c^	Control	Control
25% kcal	10	AS–G^b^	AS–G	AS–M^d^	AS–M	AS–M

^a^Control G: 10% kcal sucrose growth diet.

^b^AS-G: 25% kcal sucrose growth diet.

^c^Control: 10% kcal sucrose mature diet.

^d^AS-M: 25% kcal sucrose mature diet.

### 2.2 Dietary composition

The standard AIN-93G (growth) and AIN-93M (mature) diets (D10012G and D10012M, respectively; Research Diets Inc., USA) were used to feed the mice in the 10% kcal sucrose group. Custom-made isocaloric diets with 25% kcal from sucrose were formulated based on the standard AIN-93G and AIN-93M diet, where polysaccharides (cornstarch and maltodextrin 10) were replaced by sucrose in order to keep the energy density, as well as the amount of protein and fat the same. The full nutritional compositions of the diets are presented in Table 2. From weaning (week 4) until week 9, mice were randomized to consume either the 10 or 25% kcal sucrose version of the AIN-93G diet. From week 9 onward till week 104 or natural death, whichever was earlier, mice were fed the corresponding versions of the AIN-93M diet.

**TABLE 2:** Compositions of the study diets.

	D10012G (AIN-93G with 10% kcal sucrose)		D16060607 (AIN-93G with 25% kcal sucrose)		D10012M (AIN-93M with 10% kcal sucrose)		D16060608 (AIN-93M with 25% kcal sucrose)	
				
	Gram %	Kcal %	Gram %	Kcal %	Gram %	Kcal %	Gram %	Kcal %
**Product**								
Protein	20	20	20	20	14	15	14	15
Carbohydrate	64	64	64	64	73	76	73	76
Fat	7	16	7	16	4	9	4	9
Total		100.0		100.0		100		100
Kcal/gm	4.00		4.00		3.85		3.85	
**Ingredient**								
Casein	200	800	200	800	140	560	140	560
L-Cystine	3	12	3	12	1.8	7	1.8	7
Cornstarch	397.486	1589.94	279.5	1178	495.692	1982.768	380.075	1520.300
Maltodextrin	132	528	100	400	125	500	100	400
Sucrose	100	400	250	1000	100	400	240.625	963
Cellulose, BW200	50	0	50	0	50	0	50	0
Soybean oil	70	630	70	630	40	360	40	360
t-BHQ	0.014	0	0.014	0	0.008	0	0.008	0
Mineral Mix S10022G	35	0	35	0	0	0	0	0
Mineral mix S10022M	0	0	0	0	35	0	35	0
Vitamin mix V10037	10	40	10	40	10	40	10	40
Choline bitartrate	2.5	0	2.5	0	2.5	0	2.5	0
FD and C yellow Dye #5	0	0	0	0	0	0	0	0
FD and C Red Dye #40	0	0	0.05	0	0	0	0	0
FD and C Blue Dye #1	0	0	0	0	0	0	0.05	0
Total	1000	4000	1000.064	4000	1000	3850	1000.058	3850

### 2.3 Primary outcomes

**FIGURE 1:**
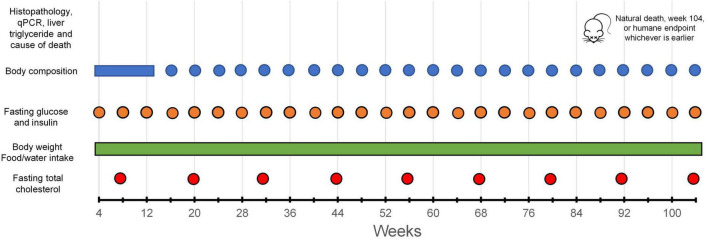
Data collection schedule.

The body weight of the mice was assessed once a week using an electronic balance (EA-300, SNOWREX International Co. Ltd., Taiwan) on the day before body composition measurement. Body composition (absolute and percentage fat mass, body fluid mass, and lean mass) was analyzed every 4 weeks or as otherwise indicated (e.g., due to public holiday, sickness, etc.), using an NMR-based body composition analyzer (Minispec Lf-50, Bruker Corporation, USA). The data collection schedule is illustrated in Figure 1. Data for these outcomes obtained after the mice started to lose >15% of body weight from the peak weight were excluded from the analysis (see Supplementary Tables 1, 2 for the *n* at each time point).

### 2.4 Secondary outcomes

#### 2.4.1 Food and water intake

Food and water intake of the mice was assessed once per week and reported as intake per day.

#### 2.4.2 Fasting blood glucose and plasma insulin

Fasting blood glucose level was measured in mice fasted for 16 h before assessment (from 5 p.m. the previous day to 9 a.m. the day of measurement). A small drop of blood was collected from the tail tip of the mice by tail pricking. Blood glucose level was measured using a commercial glucometer (AccuChek Performa, Roche Diabetes Care, Inc., USA). Additional blood was also collected from the tail to measure fasting plasma insulin level using a high-sensitivity commercial ELISA kit (Catalog #: 90080; Crystal Chem, USA). This method of blood collection ensures that blood could be drawn regularly throughout the whole experimental period/lifespan of the mice without the need for prolonged recovery of the mice.

#### 2.4.3 Fasting plasma total cholesterol and liver triglyceride (TG) content

Total cholesterol level in plasma was measured using Stanbio Cholesterol Liquicolor (Catalog #: 1010-225; Stanbio Laboratory, USA). Liver TG content was assessed with Triglyceride Colorimetric Assay Kit (Catalog #: 2200-225; Cayman Chemical, USA).

#### 2.4.4 Gene expression analysis/mouse carcass dissection

After reaching the experimental humane endpoint (weight loss of ≥15% of peak body weight, or at week 104, whichever is earlier), the mice were euthanized by injecting 50 mg/kg body weight pentobarbitone intraperitoneally. Liver samples were collected, snap-frozen, and used for gene expression analysis by real-time polymerase chain reaction (RT-PCR). The details of the experimental procedures could be found in the Supplementary text. The sequences of primers (Centre for Genome Sciences, The University of Hong Kong) used were listed in Table 3. After RT PCR amplification, a melt curve was included to confirm the specificity of the amplicon. The 2^–ΔΔCT^ method was utilized to calculate the relative gene expression (35). The sample size for the four genes examined differed due to deaths not immediately discovered (thus disallowing timely tissue extraction and storage for RT-PCR).

**TABLE 3:** Primer sequences of target proteins used in real-time PCR mRNA analysis.

Protein name	Abbreviation	Oligo name	Size	Sequence (5′ to 3′)
Actin	*Actin*	mACTINm422-F mACTINm496-R	74	AACCGTGAAAAGATGACCCAGAT CACAGCCTGGATGGCTACGT
Fatty acid synthase	*Fasn*	mFasn-m7703-F mFasn-m7904-R	201	CTCCGTGGACCTTATCACTA CTGGGAGAGGTTGTAGTCAG
Glycogen synthase	*Gys3*	mGys3-m681-F mGys3-m740-R	60	TCCTCACCACCTGGTTCCT CCACATACGGCTTCTCTTCG
Glucose-6-phosphatase	*G6pc*	mG6pc-m153-F mG6pc-m737-R	484	GGTTCATCCTTGTGTCTGTGATTG AATGCCTGACAAGACTCCAGCC
Phosphoenolpyruvate carboxykinase	*Pck1*	mpepk-m195-F mpepk-m770-R	575	AGCCTCGACAGCCTGCCCCAGG CCAGTTGTTGACCAAAGGCTTTT

#### 2.4.5 Liver histopathology

Liver samples collected were fixed with formalin, and embedded in paraffin. The sections (4 μm) were stained with hematoxylin-eosin (H&E). The stained sections were observed under bright field mode with Nikon 80i Fluorescent Microscope (Nikon DS-Ri2 camera, Nikon, Japan).

#### 2.4.6 Life expectancy

Mice were kept from week 4 until their natural death or week 104, whichever is earlier. Life expectancy was calculated using weeks of survival for each mouse. Mice which died of non-diet related ailments, e.g., acute injury or infectious diseases were recorded as censored death (*n* = 1 in the 25% kcal group), and not included in the survival analysis.

### 2.5 Power calculation

The number of mice used in each group (i.e., *n* = 10) allowed us to detect an effect size of 1.32 with 80% power and an α of 0.05 in pair-wise comparisons. Although the effect size appeared large, it should be noted that this represents a difference over the lifespan of the mice. Therefore it should be interpreted differently from the usual interpretation in shorter-term studies, as smaller effect sizes may not be considered clinically relevant over the lifespan. Based on *post hoc* analysis, an effect size of 1.32 translates to approximately a 10% difference in body weight over the whole life course, and any difference less than that is unlikely to be considered clinically meaningful (36).

### 2.6 Statistical analysis

Data were presented as mean ± SD. The normality of the continuous variables was confirmed using the Q-Q plot. GraphPad Prism 8.0.2 (GraphPad Software Inc., USA) was used to perform the statistical analyses. Kaplan-Meier survival curve was used to compare survival patterns between the two groups. A linear mixed model was used for the pairwise comparisons of time-series data of mice as it can handle missing data, and the number of observations available for each variable at each time point was available as Supplementary Tables 1, 2. One-way ANOVA was used to examine differences in relative fold change in the level of gene expression, liver triglyceride content as well as total plasma cholesterol between the 10 and 25% kcal sucrose groups. All investigators except the biostatisticians were aware of the group allocation of the experiment. A two-tailed *p* < 0.05 was considered statistically significant.

## 3 Results

**FIGURE 2:**
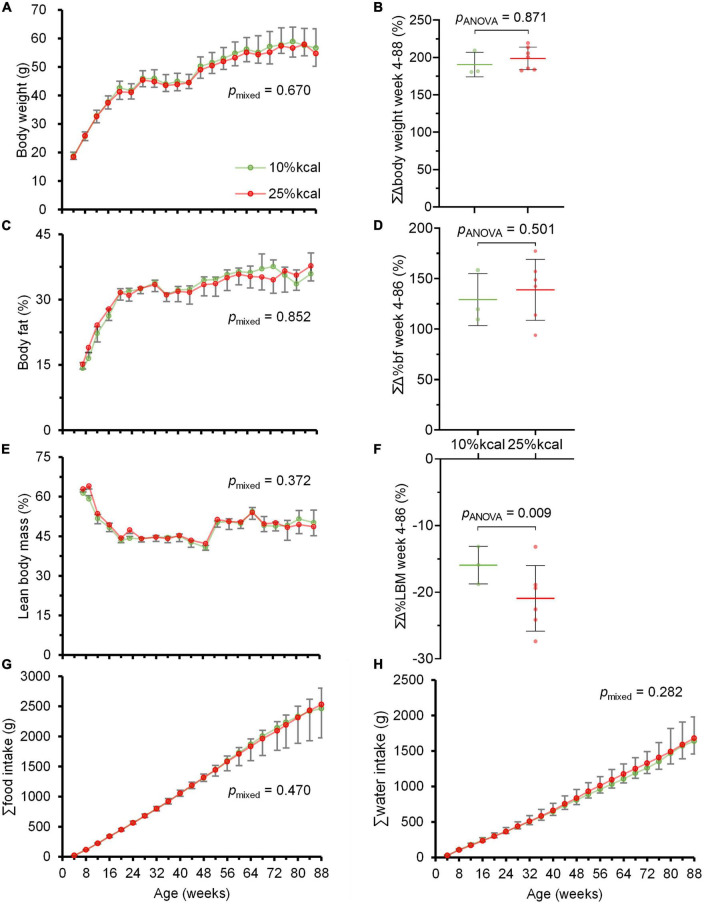
Chronic higher added sugars intake does not lead to overweight and obesity in male C57BL/6N mice. **(A)** Body weight (g); **(B)** cumulative change in body weight (%); **(C)** body fat (%); **(D)** cumulative change in body fat (%); **(E)** lean body mass (%); **(F)** cumulative change in lean body mass (%); **(G)** cumulative food intake (g); and **(H)** cumulative water intake (g) of male C57BL/6N (*n* = 10 in each group at the start, see Supplementary Tables 1, 2 for *n* at each timepoint) fed 10 vs. 25% kcal sucrose diets between weeks 4 and 88. Values are presented as mean ± SD. Differences between groups were tested using linear mixed model for time-series analysis, and one-way ANOVA for single timepoint analysis. Although the study ended at week 104, only data until week 88 were plotted as the study mice were losing weight due to aging and other ailments rather than the study diets.

**FIGURE 3:**
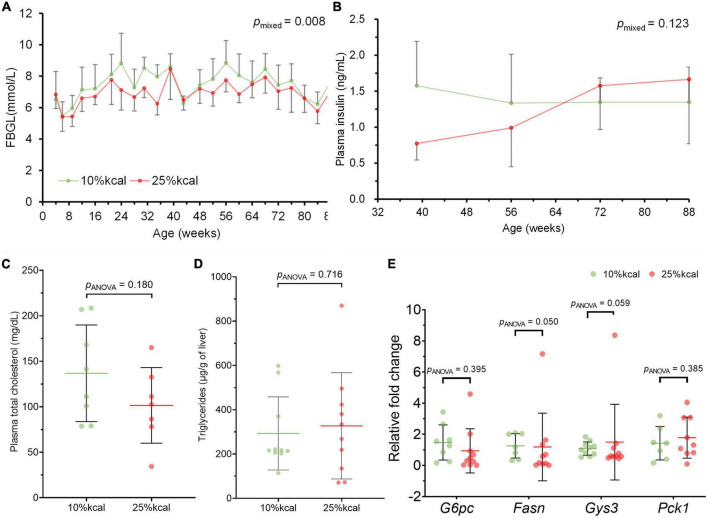
Intake of 25% kcal sucrose diet does not cause hyperglycemia and dyslipidemia and disrupt expression of related genes in male C57BL/6N mice, compared with a 10% kcal sucrose. **(A)** Fasting blood glucose level (FBGL; *n* = 10 in each group at the start, see Supplementary Table 1 for *n* at each timepoint), **(B)** fasting plasma insulin level (*n* = 9 in each group for weeks 39, 56, and 72; *n* = 3 in each group at week 88); and **(C)** fasting plasma total cholesterol (*n* = 10 in each group); **(D)** liver triglycerides (*n* = 8 in 10% kcal and 7 in 25% kcal); and **(E)** hepatic expression of *G6pc* (*n* = 8 in 10% kcal and 10 in 25% kcal), *Fasn* (*n* = 7 in 10% kcal and 9 in 25% kcal), *Gys3* (*n* = 10 in each group), and *Pck1* (*n* = 7 in 10% kcal and 8 in 25% kcal) of male C57BL/6N at time of sacrifice (week 87–104). Values are presented as mean ± SD. Differences between groups were tested using linear mixed model for time-series analysis, and one-way ANOVA for single timepoint analysis. RFC, relative fold change.

Throughout the study period, no statistically significant difference was observed in body weight, as well as body fat and lean body mass percentage between mice in the 10 and 25% kcal groups (Figures 2A–F). There were also no significant differences in cumulative food and water intake between the 10 vs. 25% kcal group (Figures 2G, H). The 25% kcal group had significantly lower fasting blood glucose levels throughout the lifespan (*p*_mixed_ = 0.008) when compared with the 10% kcal group (Figure 3A). For fasting plasma insulin levels (Figure 3B), there was no significant difference between 10 and 25% kcal mice over the lifespan (*p*_mixed_ = 0.123). For outcomes related to lipids, we found no significant differences in plasma total cholesterol level between the 10 vs. 25% kcal group (*p*_ANOVA_ = 0.180; Figure 3C). For liver TG content, there were also no significant differences between the 10 and 25% kcal groups (*p*_ANOVA_ = 0.716; Figure 3D).

There were no significant differences in gene expression for glucose 6-phosphatase (*G6pc*), glycogen synthase (*Gys3*) and phosphoenolpyruvate carboxykinase (*Pck1*) between the 10 and 25% kcal groups (Figure 3E); and lower expression of fatty acid synthase (*Fasn*) gene was observed in the 25% kcal group compared with the 10% kcal group (*p*_ANOVA_ = 0.050).

**FIGURE 4:**
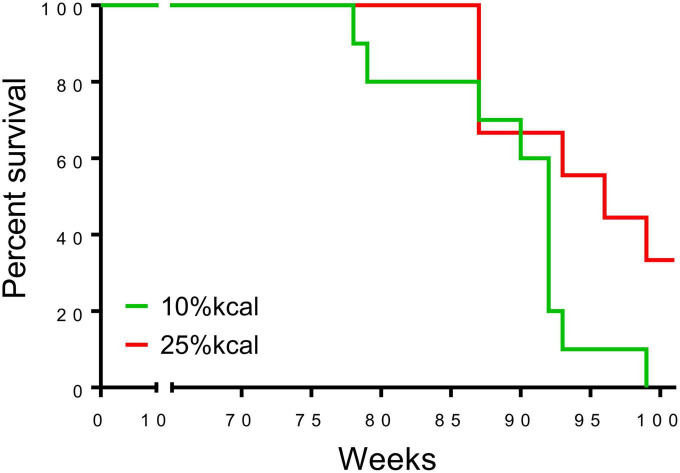
Survival curve of male C57BL/6N mice (*n* = 10 in each group) that have been fed with different diets. A censored death at 37 in the 25% kcal group was excluded from the analysis.

**FIGURE 5:**
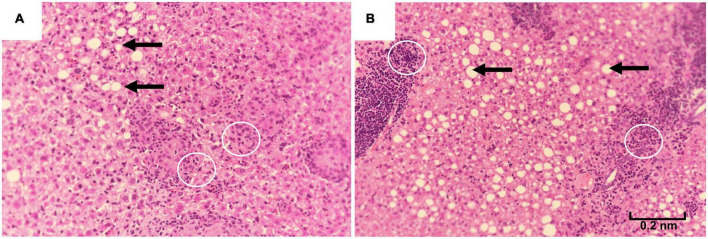
Liver histopathology of male C57BL/6N mice fed different diets at time of sacrifice, assessed using hematoxylin-eosin staining of liver samples (magnification 200 ×). **(A)** A total of 10% kcal, **(B)** 25% kcal. Both groups showed fatty lipid droplets (arrows). Prescence of Kupffer cells (white circles) were also observed.

The first deaths of the study mice were observed at 78 weeks in the 10% kcal group (mean ± SD lifespan = 89.4 ± 6.5 weeks), while mice in the 25% kcal group had a non-statistically significant higher average life span (first death at 87 weeks; mean ± SD lifespan = 94.7 ± 6.3 weeks; *p*_ANOVA_ = 0.09). Nonetheless, the survival analysis suggests that the level of sugar in the diet may not be dose-dependently related to the mortality of male C57BL/6N mice (Figure 4; *p*_survival_ = 0.106). H&E staining of liver tissue (200 ×) showed lipid accumulation in both groups, with both samples of the 25% kcal group exhibiting lipid droplets (Figure 5).

## 4 Discussion

In our study, we showed that lifelong consumption of a diet consisting of 25% kcal from sucrose generally had no significant impact on inducing overeating and obesity and most related outcomes (blood glucose and insulin level; plasma total cholesterol and TG level; liver histopathology and gene expression; and life expectancy), compared to a 10% kcal sucrose diet. To the best of our knowledge, there are no previous studies that examined the health effects of chronic higher sugar intake on metabolic and endocrine health. The *null* findings of our study thus disagree with conclusions from previous animal studies that high sugar intake causes obesity and related complications. Our data presented here suggest further pre-clinical and human studies to elucidate and confirm the effects of chronic intake of sucrose at human-relevant levels are required to better inform the dietary guidelines and public health policy development on sugar consumption.

Generally, throughout this study, we observed no significant difference in cumulative food intake between male C57BL/6N mice fed 10 vs. 25% kcal sucrose diets, which concur with the lack of significant difference in weekly mean body weight and percentage cumulative change in body weight. Our results seem to be similar to that of Ruff et al. (17), who reported that a 25% kcal sugar (as 1:1 fructose:glucose) diet for >26 weeks did not affect body weight. The study by Bouwman et al. (21) also showed that partial or complete replacement of glucose with fructose did not lead to overeating and weight gain in mice on a moderately high-fat diet, and high fructose intake at up to 30% daily kcal did not lead to overeating and weight gain in healthy subjects compared to controls with NAFLD (23). However, since body weight is not an accurate indicator of obesity, we also assessed the body composition of the mice regularly, and overall found no significant differences between groups over the whole life span of the mice. Similarly, lifelong higher consumption of sucrose in solid form did not induce overeating and obesity, which contradicts previous findings (37, 38). Of note is that most studies linking high sugar intake to obesity examined sugar in the liquid form (39, 40). It has been suggested that sugar fed in the liquid form might lead to overeating due to incomplete energy intake compensation at subsequent meals, an effect not observed for sugar intake from solid foods (41). Our *null* finding suggests the current public health focus on sugar reduction which treats all sources of sugar as equally detrimental to health may need to be re-evaluated (8).

Overall, we also found no significant differences in fasting blood glucose levels between groups across different life stages. Nevertheless, other animal studies conclude that a high-sugar diet could lead to hyperglycemia. For example, Huang et al. (42) found that rats fed a 60% w/w fructose diet, developed hyperglycemia when compared with the control group in only 8 weeks. It should be noted that these studies used supraphysiological doses to induce adverse health effects in a short period, which does not resemble normal human consumption levels in real life (43).

Gene expression analyses showed that the expression of key genes in glucose metabolism, including *G6pc* (the rate-limiting enzyme in gluconeogenesis), *Pck1* (an important enzyme in gluconeogenesis), and *Gys3* (the rate-limiting enzyme in glycogen synthesis) did not support the notion that 25% kcal sucrose consumption adversely affects glucose and lipid metabolism. The finding concurs with the non-significant changes in the fasting insulin and blood glucose levels between groups. In fact, the effect of a high-sugar diet on plasma insulin level is somewhat controversial, with some researchers finding that plasma insulin level was increased after feeding a high-sugar diet, and others obtaining *null* findings (42, 44). This might be caused by the different study durations and sugar dosages in the diet. Nevertheless, our data clearly demonstrated that even life-long consumption of a diet with 25% kcal sucrose did not appear to have altered glucose metabolism in mice.

We also observed no significant adverse effects on lipid metabolism (plasma total cholesterol and liver TG content) with the higher sugar diet. Although liver histopathology examination in our study showed that the 25% kcal sucrose group had more liver lipid droplets than the 10% kcal group and that fatty liver was consistently observed in the 25% kcal sucrose mice, several mice from the 10% kcal group also developed mild fatty liver, in addition to the presence of Kupffer cells, which is a sign of hepatic inflammation. Co-presence of Kupffer cells and lipid droplets is suggestive of NAFLD (45). Nonetheless, the number of lipid droplets observed in our study is small compared to true NAFLD developed earlier in the life cycle, suggesting that the presence of liver lipid droplets and Kupffer cells is more likely caused by aging than the diet itself (46). Our findings agree with that of Bouwman et al. (21), which also found a lack of significant effect of partial or complete replacement of glucose with fructose on liver TG levels in mice. However, other studies have found that sugar consumption at supraphysiological doses increased fasting plasma cholesterol levels in mice/rats (17).

Discrepancies in levels of liver TG content observed in our study and that of others could be explained by several factors. First, our study investigated sucrose, whereas most relevant animal studies administered fructose as the dietary treatment. Sucrose and fructose are absorbed and metabolized differently in humans (13, 47). While fructose can bypass metabolic regulation, the metabolism of sucrose is somewhat regulated in the liver, which reduces metabolic and endocrine disturbances compared to consuming fructose alone (48).

Although mice in the 25% kcal group had a non-statistically significant higher average life span than those in the 10% kcal group, the survival analysis suggests that the level of sugar in the diet may not be dose-dependently related to the mortality of male C57BL/6N mice. This indicates that lifelong consumption of a higher-sugar diet may not adversely affect longevity compared to a lower-sugar diet. This is in stark contrast to the findings of Ruff et al. (17) who reported chronic consumption of a 1:1 mixture of fructose and glucose at 25% kcal induced higher mortality in female wild mice but not male mice, in addition to results from studies conducted in *C. elegans* and *D. melanogaster*, which suggest that high sucrose/fructose diet may reduce their lifespan, while intake at low levels could prolong it (18, 19). Since our study utilized only male C57BL/6N mice, the potential sex difference in the effect of sugar on mortality should be further investigated. Nonetheless, we believe our *null* findings on mortality are not surprising considering that lifelong consumption of a higher sugar diet and diet switching had no significant influence on most metabolic and endocrine parameters assessed.

Our study has several strengths compared to previously published studies in this field of research. First, it is the first of its kind to examine the effect of lifelong higher sucrose consumption on metabolic and endocrine outcomes. Second, unlike previous studies, the type and dosages of sugar (i.e., sucrose) used in this study resemble real-life human consumption levels (43). Third, we used a 10% kcal sucrose diet as the control group, which allows for a better translation of conclusions into the human situation, because in reality, humans rarely consume a diet devoid of sugars (43). Last, regular data collection throughout the whole lifecycle enabled us to gain a comprehensive understanding of the interaction between the higher sugar diet and metabolism over different life stages. Findings from this study provided the first hint that sugar consumption at human-relevant levels may not be as detrimental to health as currently portrayed by the media and in the literature, which calls for more large-scale epidemiological studies conducted in populations with various genotypes and eating habits to investigate the long-term health impact of high sugar consumption in humans.

Several limitations of this study should also be considered when interpreting the results. First, only *Fasn*, *G6pc*, *Gys3*, and *Pck1* were measured in our gene expression analysis. Although these genes code for important regulators of carbohydrate and lipid metabolism, there are more genes that also affect the relevant metabolic processes, such as glucose transporters 1 and 4 (*Slc2a1*/GLUT 1 and *Slc2a4/*GLUT 4) and acetyl CoA carboxylase (*Acaca*/ACC). Nonetheless, considering that there were generally no significant differences between groups in the phenotypic outcomes of interest, results from additional assays on these genes are not expected to cause a material difference in the conclusions. Second, the 10% kcal group consumed a diet with some sugar replaced by cornstarch and maltodextrin, which is rapidly absorbed in the small intestine (49). Although it has been shown that long-term intake of maltodextrin and cornstarch may have adverse health effects such as impaired insulin sensitivity and hyperlipidemia (50), the difference in % kcal of glucose from digestion of cornstarch and maltodextrin between 10 and 25% kcal sucrose diets in our study is considered small (e.g., 55 vs. 47.5% kcal glucose for 10% and 25% kcal sucrose diets, respectively, assuming 60% kcal from carbohydrates), that would only have minute influences if any on our study results, which overall also showed no significant differences between groups. Third, fecal energy density was not measured in our study, however, since both sucrose and starch are digested and absorbed quickly, and *null* findings were observed for most outcomes, this should not have a major influence on validity of our study conclusions. Additionally, most of the study mice also started to lose weight due to aging rather than the assigned diet at the end of the study period, which may have an impact on the outcomes of interest, although both groups were affected similarly. The small number of mice used in each group might also limit the statistical power to detect difference in the outcomes of interest, however, since the mean values did not differ at most time points examined, the difference between groups, if any, are expected to be small and clinically irrelevant. Finally, only male mice were included in this study, which could impair the translatability of the study conclusions to human populations of both sexes, although female mice were not included in order to minimize the effect of hormone fluctuations on study outcomes. Future studies should examine the effect of a chronic high-sugar diet on endocrine and metabolic health in both sexes.

## 5 Conclusion

Our study showed that a higher sugar diet at human-relevant levels did not induce overeating and obesity, impaired glucose and lipid metabolism, or lower life expectancy, compared to a lower sucrose diet in mice, and dietary intervention at different life stages had no effect on the health outcomes of interest. Our results disagree with conclusions from previous animal studies that high sugar intake causes obesity and related complications. Future research should examine the intergenerational effect of chronic high sugar consumption.

## Data availability statement

Data for this study are available upon reasonable request. Interested parties should contact the corresponding author JL, jimmyl@hku.hk for access.

## Ethics statement

This animal study was reviewed and approved by the Committee on the Use of Live Animals for Research, The University of Hong Kong.

## Author contributions

JW, EL, CC, and JL: conceptualization and funding acquisition. MT, CC, and JL: methodology and supervision. YZ and JX: formal analysis. RY, VC, and TH: investigation. CC and JL: resources. RY, VC, TH, and JL: data curation and writing—original draft preparation. RY, IT, WS, JW, EL, CC, and JL: writing—review and editing. RY, VC, and JL: visualization. JL: project administration. All authors have read and agreed to the published version of the manuscript.
