# Supplementary material for: Effect of lifelong sucrose consumption at human-relevant levels on food intake and body composition of C57BL/6N mice
Source: Front Nutr. 2022 Dec 15;9:1076073. doi: 10.3389/fnut.2022.1076073 (PMC9798237; doi:10.3389/fnut.2022.1076073)
Supplement: Supplementary file 1 [file Image_1.pdf]

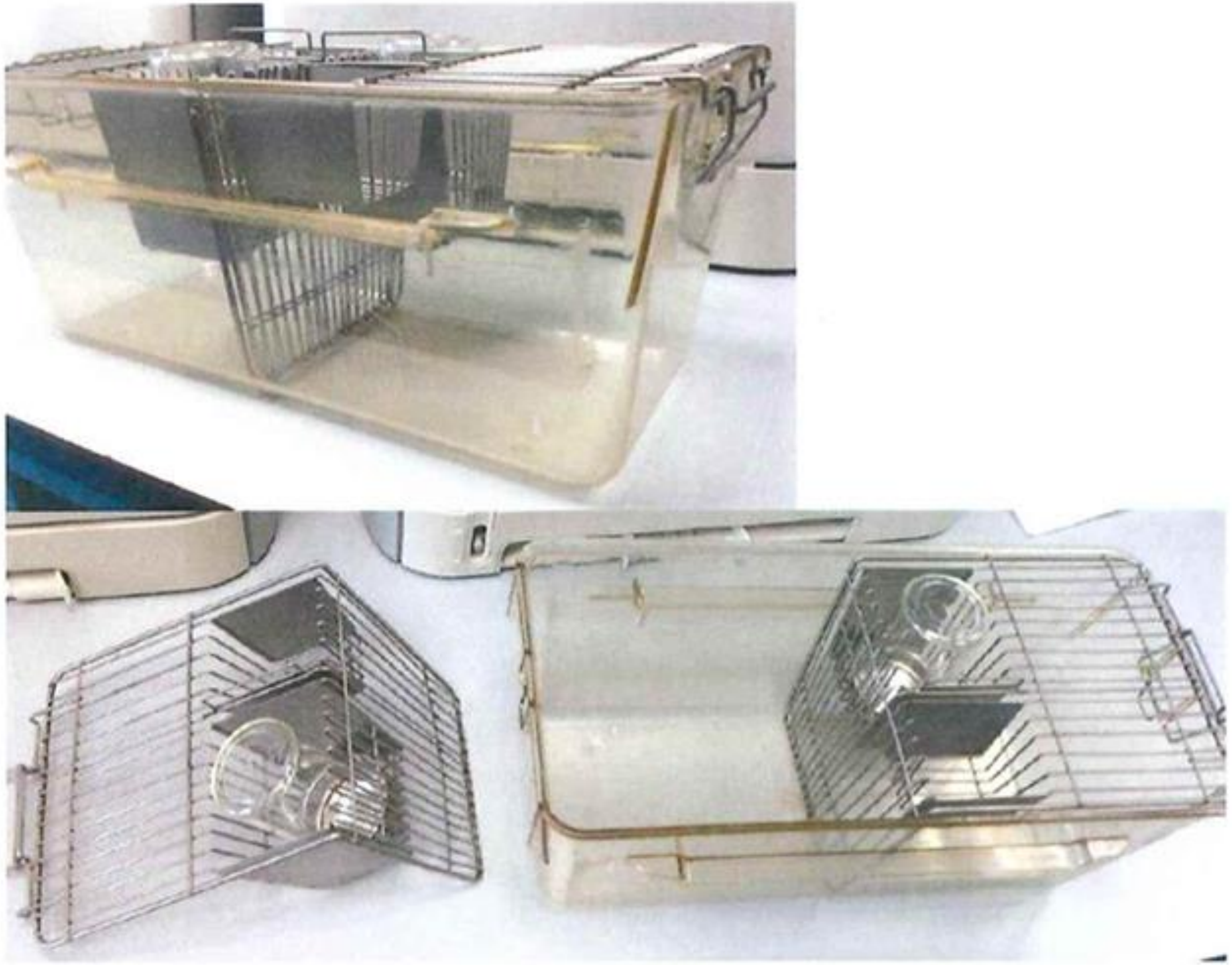

Online Supplemental Fig. 1 – Special cage cover that separates the cage in half for individual food and water intake assessment
