# Supplementary material for: Effect of lifelong sucrose consumption at human-relevant levels on food intake and body composition of C57BL/6N mice
Source: Front Nutr. 2022 Dec 15;9:1076073. doi: 10.3389/fnut.2022.1076073 (PMC9798237; doi:10.3389/fnut.2022.1076073)
Supplement: Supplementary file 2 [file Table_1.DOCX]

**Supplementary Text** – Experimental procedures for gene expression analysis

Total RNA was extracted from approximately 0.1 g of liver tissue using TRI Reagent® (Catalogue #: TB 126; Molecular Research Center, Inc., USA) according to manufacturer’s instructions. The concentrations of RNA were adjusted to 800-1700 ng/*µ*L with diethyl pyrocarbonate (DEPC) water in each sample, which were stored at -80°C until further analysis. Reverse transcription was carried out with 5 *µ*g of RNA. The RNA samples and nuclease-free water were first denatured again in the thermal cycler (iCycler, Bio-Rad, USA) under 65°C for 10 minutes. After that, 20 *µ*L of cDNA for each sample were produced using the iScript^TM^ Advanced cDNA synthesis kit (Catalogue #: 1725038; Bio-Rad, USA). Reactions were incubated at 42°C for 30 min for reverse transcription and 95°C for 1 min to inactivate the reverse transcriptase. The cDNA was stored at -40°C until further analysis. RT PCR was performed using StepOnePlus RT PCR System (Applied Biosystems, USA). iTaq™ Universal SYBR® Green Supermix (Catalogue #: 1725121; Bio-Rad, USA) was used as reagent for detection. Each amplification mixture (10 *µ*L) contained 1 *µ*L cDNA, 0.4 *µ*L primers, 3.6 *µ*L water and 5 *µ*L Universal SYBR Green Supermix. One sample was prepared for each mouse.
