# Supplementary material for: Effect of lifelong sucrose consumption at human-relevant levels on food intake and body composition of C57BL/6N mice
Source: Front Nutr. 2022 Dec 15;9:1076073. doi: 10.3389/fnut.2022.1076073 (PMC9798237; doi:10.3389/fnut.2022.1076073)
Supplement: Supplementary file 3 [file Table_2.DOCX]

**Online Supplemental Table 1 –** Sample size for body weight, food and water intake, and blood glucose level measurement at each time point

| **Weeks** | **Control** | **HS** |
| --- | --- | --- |
| 5 | 10 | 10 |
| 6 | 10 | 10 |
| 7 | 10 | 10 |
| 8 | 10 | 10 |
| 9 | 10 | 10 |
| 10 | 10 | 10 |
| 11 | 10 | 10 |
| 12 | 10 | 10 |
| 13 | 10 | 10 |
| 14 | 10 | 10 |
| 15 | 10 | 10 |
| 16 | 10 | 10 |
| 17 | 10 | 10 |
| 18 | 10 | 10 |
| 19 | 10 | 10 |
| 20 | 10 | 10 |
| 21 | 10 | 10 |
| 22 | 10 | 10 |
| 23 | 10 | 10 |
| 24 | 10 | 10 |
| 25 | 10 | 10 |
| 26 | 10 | 10 |
| 27 | 10 | 10 |
| 28 | 10 | 10 |
| 29 | 10 | 10 |
| 30 | 10 | 10 |
| 31 | 10 | 10 |
| 32 | 10 | 10 |
| 33 | 10 | 10 |
| 34 | 10 | 10 |
| 35 | 10 | 10 |
| 36 | 10 | 10 |
| 37 | 10 | 10 |
| 38 | 10 | 9* |
| 39 | 10 | 9 |
| 40 | 10 | 9 |
| 41 | 10 | 9 |
| 42 | 10 | 9 |
| 43 | 10 | 9 |
| 44 | 10 | 9 |
| 45 | 10 | 9 |
| 46 | 10 | 9 |
| 47 | 10 | 9 |
| 48 | 10 | 9 |
| 49 | 10 | 9 |
| 50 | 10 | 9 |
| 51 | 10 | 9 |
| 52 | 10 | 9 |
| 53 | 10 | 9 |
| 54 | 10 | 9 |
| 55 | 10 | 9 |
| 56 | 10 | 9 |
| 57 | 10 | 9 |
| 58 | 10 | 9 |
| 59 | 10 | 9 |
| 60 | 10 | 9 |
| 61 | 10 | 9 |
| 62 | 10 | 9 |
| 63 | 10 | 9 |
| 64 | 10 | 9 |
| 65 | 10 | 9 |
| 66 | 10 | 9 |
| 67 | 10 | 9 |
| 68 | 10 | 9 |
| 69 | 10 | 9 |
| 70 | 10 | 9 |
| 71 | 10 | 9 |
| 72 | 9 | 9 |
| 73 | 9 | 9 |
| 74 | 9 | 8 |
| 75 | 9 | 8 |
| 76 | 9 | 8 |
| 77 | 9 | 8 |
| 78 | 8 | 8 |
| 79 | 7 | 8 |
| 80 | 7 | 8 |
| 81 | 7 | 7 |
| 82 | 7 | 7 |
| 83 | 7 | 7 |
| 84 | 7 | 7 |
| 85 | 5 | 7 |
| 86 | 5 | 7 |
| 87 | 4 | 6 |
| 88 | 3 | 6 |
| 89 | 3 | 6 |
| 90 | 3 | 6 |
| 91 | 3 | 6 |
| 92 | 2 | 5 |
| 93 | 0 | 4 |
| 94 | 0 | 4 |
| 95 | 0 | 4 |
| 96 | 0 | 4 |
| 97 | 0 | 3 |
| 98 | 0 | 2 |
| 99 | 0 | 2 |
| 100 | 0 | 2 |
| 101 | 0 | 2 |

*censored death in survival analysis

The number of mice at each time point differs from the survival analysis. Values obtained after the mice experience more than 15% weight loss from the peak value were removed as these are not representative of the dietary effect. Blood glucose level measured every 4 weeks from week 4.
