# Supplementary material for: Effect of lifelong sucrose consumption at human-relevant levels on food intake and body composition of C57BL/6N mice
Source: Front Nutr. 2022 Dec 15;9:1076073. doi: 10.3389/fnut.2022.1076073 (PMC9798237; doi:10.3389/fnut.2022.1076073)
Supplement: Supplementary file 4 [file Table_3.DOCX]

**Online Supplemental Table 2 –** Sample size for %body fat and lean body mass measurements at each time point

| **Weeks** | **Control** | **HS** |
| --- | --- | --- |
| 7 | 10 | 10 |
| 9 | 10 | 10 |
| 12 | 10 | 10 |
| 16 | 10 | 10 |
| 20 | 10 | 10 |
| 23 | 10 | 10 |
| 27 | 10 | 10 |
| 32 | 10 | 10 |
| 36 | 10 | 9* |
| 40 | 10 | 9 |
| 44 | 10 | 9 |
| 49 | 10 | 9 |
| 53 | 10 | 9 |
| 57 | 10 | 9 |
| 61 | 10 | 9 |
| 65 | 10 | 9 |
| 69 | 9 | 9 |
| 73 | 9 | 9 |
| 77 | 9 | 8 |
| 81 | 7 | 7 |
| 86 | 5 | 7 |
| 89 | 3 | 6 |
| 93 | 0 | 4 |
| 96 | 0 | 4 |
| 100 | 0 | 2 |

*censored death

The number of mice at each time point differs from the survival analysis. Values obtained after the mice experience more than 15% weight loss from the peak value were removed as these are not representative of the dietary effect.
